# Functional SNP panel for parentage assessment and assignment in worldwide goat breeds

**DOI:** 10.1186/s12711-018-0423-9

**Published:** 2018-11-19

**Authors:** Andrea Talenti, Isabelle Palhière, Flavie Tortereau, Giulio Pagnacco, Alessandra Stella, Ezequiel L. Nicolazzi, Paola Crepaldi, Gwenola Tosser-Klopp, AdaptMap Consortium

**Affiliations:** 1 Dipartimento di Medicina Veterinaria, Università degli Studi di Milano, Via Celoria 10, 20133 Milan, Italy; 2 GenPhySE, INRA, Université de Toulouse, INPT, ENVT, 31326 Castanet Tolosan, France; 3 Fondazione Parco Tecnologico Padano (PTP), Via Einstein, Cascina Codazza, 26900 Lodi, Italy

## Abstract

**Background:**

International standard panels of single nucleotide polymorphisms (SNPs) have replaced microsatellites in several species for parentage assessment and assignment (PA) purposes. However, such a resource is still lacking in goats. The application of a cheap tool for PA would help the management of goat populations by improving the reliability of pedigree registration and, consequently, allow a better implementation of breeding schemes or conservation programs.

**Results:**

Using data from the current GoatSNP50 chip, starting from a worldwide dataset of more than 4000 animals belonging to more than 140 breeds and populations from the AdaptMap initiative, we selected a panel of 195 SNPs. The assignment rate of this panel was up to 100% on an additional dataset that included 2000 Alpine and Saanen animals and highly related candidate sires.

**Conclusions:**

In this study, we defined a highly informative SNP panel, which will be publicly available to worldwide breeders and laboratories. Its development on such a large number of breeds and populations, together with validation on a second set of cosmopolitan breeds, makes it a promising and important genomic tool for the goat species.

**Electronic supplementary material:**

The online version of this article (10.1186/s12711-018-0423-9) contains supplementary material, which is available to authorized users.

## Background

Reliable genealogical information is essential for accurate genetic evaluation and the success of genetic improvement programs. Such goals can be achieved either by managing individual mating or by using artificial insemination, strategies that are time-consuming, expensive and sometimes difficult to incorporate into existing management practices such as summer pasturing in open areas and collective nurseries of kids. An approach to overcome this limitation is to determine genealogy after birth by a parentage tool that determines either both the dam and sire for a kid or only the sire (if the dam is recorded reliably). In sheep flocks using natural mating, a recent study [1] showed that this can lead to an improved annual genetic gain.

There are about 1 billion goats in the world, with ~ 1000 breeds [2]. Goats are genetically diverse, adapted to various landscapes, climates, and raised for meat, milk or fiber. They are quite easy to breed including in intensive rearing conditions and in the harsh conditions of developing countries [3]. Thus, although the design of an international parentage-testing tool is challenging, it is the best way to deliver a cheap and widely available tool for species with a comparatively low economic value.

Molecular markers have been used for decades for this purpose in goat [4, 5], for which the International Society for Animal Genetics (ISAG) recommends the use of a validated panel of microsatellites for such analyses [6].

The recent availability of high-throughput sequencing technologies allowed the production of dense single nucleotide polymorphism (SNP) chips. In goat, the identification of ~ 12 million SNPs led to the design of a 53 K SNP chip, the GoatSNP50 chip, which is extensively used across the world [7]. SNPs have many advantages, which explain why they are replacing microsatellites in several parentage marker sets [8–10]. In goats, a recent study proposed a method to select SNPs for this purpose, even when using only a few breeds and animals [10].

The AdaptMap initiative (www.goatadaptmap.org; [11]) has collated 53 K genotypes from more than 140 breeds from 17 countries, providing an international dataset. Starting from AdaptMap SNP data, our aim was to select a panel of SNPs with high performance for both parentage assessment and assignment for 3887 animals belonging to 91 breeds.

Two approaches were used: first, a method based on detecting the SNPs that best distinguish related pairs of individuals from all pairwise comparisons (relationship-based procedure), and second, a method that selects the SNPs that maximize the minor allele frequencies (MAF) in the largest number of populations (MAF-based procedure), respectively.

## Methods

### Preparation of a shared dataset

The starting dataset for this study was produced under the scope of the AdaptMap initiative. After initial quality control filtering (based on the following criteria: SNP call rate higher than 98%, animal call rate higher than 96%, removal of animal duplicates that had a proportion of genotypes identical-by-state (IBS) higher than 99%) [12], the dataset included 4261 individuals from 156 breeds and 48,895 SNPs from the Illumina GoatSNP50 chip [7]. Crossbred animals and populations with less than 15 individuals were removed. Cosmopolitan breeds were considered at the country level and thus split into different populations (e.g. Alpine breed from Switzerland, France and Italy were considered separately). A total of 106 populations were considered in the subsequent analyses, and all unmapped SNPs, according to the ARS1 SNP map version (http://www.goatgenome.org/projects.html) [13], were removed.

After data screening, we proceeded with the construction of the SNP panel for parentage assessment. We applied two methods that aim at detecting different subsets of SNPs: (i) a relationship-based procedure that detects the SNPs that best distinguish between related and unrelated pairs of animals; and (ii) a minimum allele frequency (MAF)-based procedure that detects the highly variable SNPs within the different populations.

### Relationship-based procedure

#### Additional dataset editing and detection of relationships

All SNPs mapping to the X chromosome were excluded from the analysis. Then, a parentage test was performed on all pairwise comparisons of individuals by the detection of discordant homozygotes (also known as mendelian errors = ME) that were calculated using an in-house script at all 46,732 autosomal SNPs. All pairs of individuals that shared a small number of ME (< 100) were considered as parent–offspring (PO, mean ME ± SD = 12.2 ± 6.2), whereas the remaining were treated as not PO (NPO, mean ME ± SD = 6561.3 ± 1206.6).

#### Selection of SNPs and parentage assessment

The most informative SNPs for parentage assessment were detected by using a newly developed procedure, based on the method for handling a large number of pairwise comparisons, published by Talenti et al. [10]. This new approach includes three steps that allows the reduction of highly complex datasets: (1) detection of the informative SNPs on each autosome; (2) detection of discriminant SNPs; and (3) reduction to the smallest panel size.

Step (1) was performed on the quality-checked dataset and consisted of a first reduction of the panel size using: the exclusion probability (Pe), i.e. the probability of observing opposing homozygotes at a SNP between a random eligible adult and a random offspring [8]; and the probability of a coincidental match (Pi), i.e. the probability of a random coincidental match at a SNP between random animals [8]) that was calculated using an in-house script. In this step, we selected SNPs that had a Pi less than 1.0 and a Pe greater than 0.0 in a maximum of seven populations in order to keep a large number of informative SNPs.

Step (2) included a recursive linear discriminant analysis (LDA) using the *MASS* package in R [14, 15]. LDA was performed on the pairwise comparisons between *N* individuals at each genotype, considering the PO/NPO labeling as the classifier. This created a dataset composed of $\frac{{\left[{N\,x\,\left({N - 1} \right)} \right]}}{2}$ rows, and columns equal to the number of SNPs. Briefly, each SNP was evaluated for genotypic concordance at each pair of individuals, with the genotypes as the number of shared alleles (0/1/2). This dataset was analyzed through LDA analysis to detect the SNPs that best distinguish PO and NPO pairs of individuals. Since the analysis includes a large number of individual comparisons (> 7 × 10^6^), we developed a procedure to compare all available PO pairs with a large number of randomly chosen subsets of NPO pairs. The number of LDA performed was equal to the number of PO pairs, and then NPO pairs equal to 10 times the number of PO pairs were randomly selected.

The best 200 SNPs resulting from the recursive LDA were considered for the subsequent analysis, with the number chosen to correspond to the size of the bovine ISAG panel [16]. After selection of the 200 best SNPs, step (3), i.e. a final reduction was performed by evaluating each SNP separately, excluding the SNPs the removal of which did not increase the number of false positives. Although the 200-SNP size is equivalent to the official ISAG panel for the cattle species, this additional reduction was done to define the smallest subset of SNPs that can be used to obtain reliable results.

### Minimum allele frequency based procedure

Because estimation of MAF is sensitive to population size and relationships between animals, a population-reduction approach was applied for populations with more than 50 animals, and highly related animals were removed based on ME [12]. By using PLINK 1.9 [17], MAF were computed for each SNP in each population. Then, two successive minimum MAF thresholds were applied to the SNPs on the 29 autosomes: the first threshold, i.e. MAF < 0.10, was applied to all 106 populations, and the second one, i.e. MAF < 0.20, was applied only to the 30 populations without related pairs of animals. For the X chromosome, SNPs were retained if they had a minimum MAF higher than 0.05 in each of the 106 populations. In the end, a linkage disequilibrium (LD)-based pruning step using PLINK’s simple pairwise model, was performed to remove SNPs that were in LD (*r*^2^ > 0.01) with each other by considering 50-kb windows and a 5-SNP step (command: –indep-pairwise 50 5 0.01). No minimum threshold for the minimum distance between markers was set.

### Performances of the joint SNP panel

The two panels, which were selected either with the relationship- or the MAF-based procedure, were pooled. Then, we calculated: (1) MAF for all populations and SNPs; (2) Pe and Pi, at each SNP and for the whole panel, for all populations; (3) LD between SNPs; (4) the performance of the joint panel in all populations; (5) the assignment rate of the joint panel; and (6) the power of sex determination of the joint panel.

The Pe and Pi statistics were estimated for each of the 106 populations to evaluate the ability of the SNPs to exclude individuals as potential parents or the probability of having a match at that genotype by chance. Finally, the P statistics for the whole panel were calculated as shown in [10]. Pe values for each population were reported as − log_10_(Pe) to facilitate interpretation of the results and for populations that had a Pe = 0, the value was set to a default value of 16. Similarly, Pi were reported as − log_10_(Pi)/10, to make them comparable to the Pe scores (see Additional file 1: Table S1). Pairwise LD between all SNPs included in the resulting 195-SNP panel was estimated using PLINK 1.9 [17].

After estimating LD levels in the whole dataset, we evaluated the exclusion ability of the panel based on the direct genotypic exclusion. In this case, a pair of individuals was defined as unrelated if they had two or more discordant homozygotes (1% of all SNPs). Two French populations from a quantitative trait locus (QTL) study [18] were used to assess the assignment efficiency of the SNP panel. The genotypes for the SNPs in this subset were extracted from 53 K genotypes for 810 Saanen and 1175 Alpine females, and highly related candidate sires: the true sires (9 and 11, respectively, in the Saanen and Alpine breeds) and half-sibs or sires of the true sires (62 and 70, respectively, in the Saanen and Alpine breeds). Genotypes were missing for all of the dams. Then, an in-house script, based on a maximum likelihood approach and exclusion [19, 20] was applied to infer paternities. A LOD score was computed as the log of the ratio between the likelihood given the genotype of both parents and the likelihood given the genotype of the dam (or allelic frequencies of the parental population if the dam was missing). For each female, all candidate sires were tested, including the true sires. The sire with the best results was kept according to the LOD score (> 0), the number of missing SNPs (< 50), the number of SNPs showing Mendelian errors (< 2) and the posterior probability of the candidate being the true sire (≥ 0.99). The sire identified from this panel was compared to the true sire, determined by the 53 K genotypes.

Two parameters that indicated the efficiency of the parentage test were computed: the specificity—as the ratio of the number of nonparents excluded over the total number of nonparents; and the sensitivity—as the ratio of true parents assigned over the total number of true parents.

### Sex determination

Because no SNPs were mapped to the Y chromosome, we used the SNPs on the X chromosome to test the probability that an animal was simultaneously homozygous at all the SNPs, and thus, the probability that a female is confused with a male. We checked that none of the SNPs mapped to the pseudo-autosomal region (data not shown). This probability was computed for each population as: $\varPi^{{N_{\text{SNP}}}} \left({f^{2}_{A} + \left({1 - f_{A}} \right)} \right)$, where $f_{A}$ is the MAF.

## Results and discussion

### Shared dataset preparation

After removing unmapped SNPs, 48,161 SNPs distributed across all chromosomes were retained in the dataset. Three hundred and seventy-four individuals belonging to 50 populations with less than 13 animals and 102 animals from 12 crossbred populations were removed. Seven cosmopolitan breeds were divided into 22 different populations based on their country of origin. The final dataset consisted of 3887 animals from 106 populations corresponding to 91 different breeds (see Additional file 1: Table S1).

### Construction of the dataset and detection of relationships

After exclusion of SNPs mapping to the sex chromosomes, 46,732 SNPs distributed over the autosomes were retained in the dataset and used to perform the relationship-based selection. A total of 7,552,441 pairwise comparisons between individuals were performed; 802 pairs of individuals were found as PO (0.01% of the total pairwise comparisons). Among the 106 populations, 76 were found with PO pairs (see Additional file 1: Table S1). The number of PO pairs per population ranged from 1 to 188, with an average of 11.

### Relationship-based selection

The first step, excluding uninformative SNPs in more than seven populations based on Pe/Pi, led to the removal of 45,553 SNPs, leaving only 1179 markers (i.e. 2.5% of all SNPs). This is the strongest selection step performed on the whole dataset, since it is difficult to identify SNPs that have a Pe greater than 0 in a large number of populations. A more stringent threshold would lead to a larger number of SNPs removed, which is not directly comparable with the ability to discriminate between PO and NPO pairs.

The second step selected a 200-SNP set, which was further reduced by removing SNPs that did not affect the number of false positives detected. This procedure reduced the size of the panel to 147 SNPs (see Additional file 2: Table S2), which performed equally well as the 200-SNP panel.

### SNP selection based on MAF

Among the 3887 animals of the shared dataset, 2854 were retained after population reduction to compute MAF (see Additional file 1: Table S1). The size of the populations ranged from 13 to 50, with an average of 27 animals per population. The selection based on MAF thresholds led to retaining 31 SNPs that mapped to the 29 autosomes and 27 SNPs that mapped to the X chromosome. Among these, eight SNPs on the X chromosome were removed after the LD-based pruning, leading to a 50-SNP panel.

### Creation of a single joint panel

The two approaches used different thresholds and methods to select the SNPs that were necessary to assess or assign parentage among individuals. This may result in the stringency of thresholds being higher in one method compared to the other, and therefore lead to the identification of panels with different sizes. The 147-SNP panel selected in the relationship-based procedure and the 50-SNP panel selected in the MAF-based procedure were pooled to create a unique panel of SNPs with either a high discrimination power or a high variability in the different populations. After removing two SNPs that were present in both panels, 195 SNPs were retained (see Additional file 2: Table S2). The presence of such a small number of shared SNPs between the two methodologies confirm that they are based on different principles, with the first one focusing on the SNPs that distinguish mainly the pairs of related and unrelated individuals and the second one identifying the SNPs that vary mainly within the different breeds. These 195 SNPs were not distributed uniformly along the genome. The largest absolute number of SNPs was on the X chromosome (*N* = 19, 1.19% of the initial SNPs on that chromosome), which were also retained for their usefulness in sex determination. Among the autosomes, the largest number of SNPs was on chromosome 22 (*N* = 13, 1.18% of the initial number of SNPs on that chromosome), whereas chromosome 16 was the only one with no SNPs left after reduction.

### Performances of the joint panel

#### Performances

One hundred and fifty-nine SNPs belonging to the joint panel had a MAF higher than 0 in all populations, 31 SNPs were monomorphic in one population, three SNPs in two populations and two SNPs in three populations. The average MAF value for each population ranged from 0.277 (Madagascar Menabe) to 0.413 (Egyptian Barki breed).

The number of populations that had no exclusion power at the identified SNPs (number of populations with Pe = 0 at a single SNP) ranged from 1 to 12 (1 to 11% of all populations), as shown in Additional file 3: Figure S1a. Among the SNPs, for 92.8% of the SNPs there were more than seven breeds with Pe = 0, as expected since this parameter is one of the major criteria used to select the SNPs during the first step of the relationship-based procedure. The average Pe for each breed ranged from 0.072 for the Pakistan Barbari breed to 0.152 for the Italian Ciociara Grigia (see Additional file 3: Figure S1b). The − log_10_(Pe) values ranged from 6.5 for the Pakistan Barbari breed to 16 for 15 breeds (Saanen, Oasis, Blanca de Rasquera, Alpine, Corse, Fosses, Poitevine, Pyrenees, Aspromontana, Ciociara Gricia, Nicastrese, Sarda, Red Sokoto and Spanish).

The number of populations that had a probability of 1 of a coincidental match at the identified SNPs (number of populations with Pi = 1 at a single SNP) ranged from 0 to 3 (0 to 3% of all populations), as shown in Additional file 3: Figure S1a. For 81% of the SNPs, there were no breeds with Pi = 1, confirming the ability to distinguish between individuals. The average Pi for each breed ranged from 0.385 for the Egyptian Barki breed to 0.516 for the Spanish Palmera breed (see Additional file 3: Figure S1b). As shown in Additional file 1: Table S1, the − log_10_(Pi)/10 values obtained on the 195-SNP panel ranged from 5.9 for the Palmera breed to 8.1 for the Saidi breed.

**Fig. 1:**
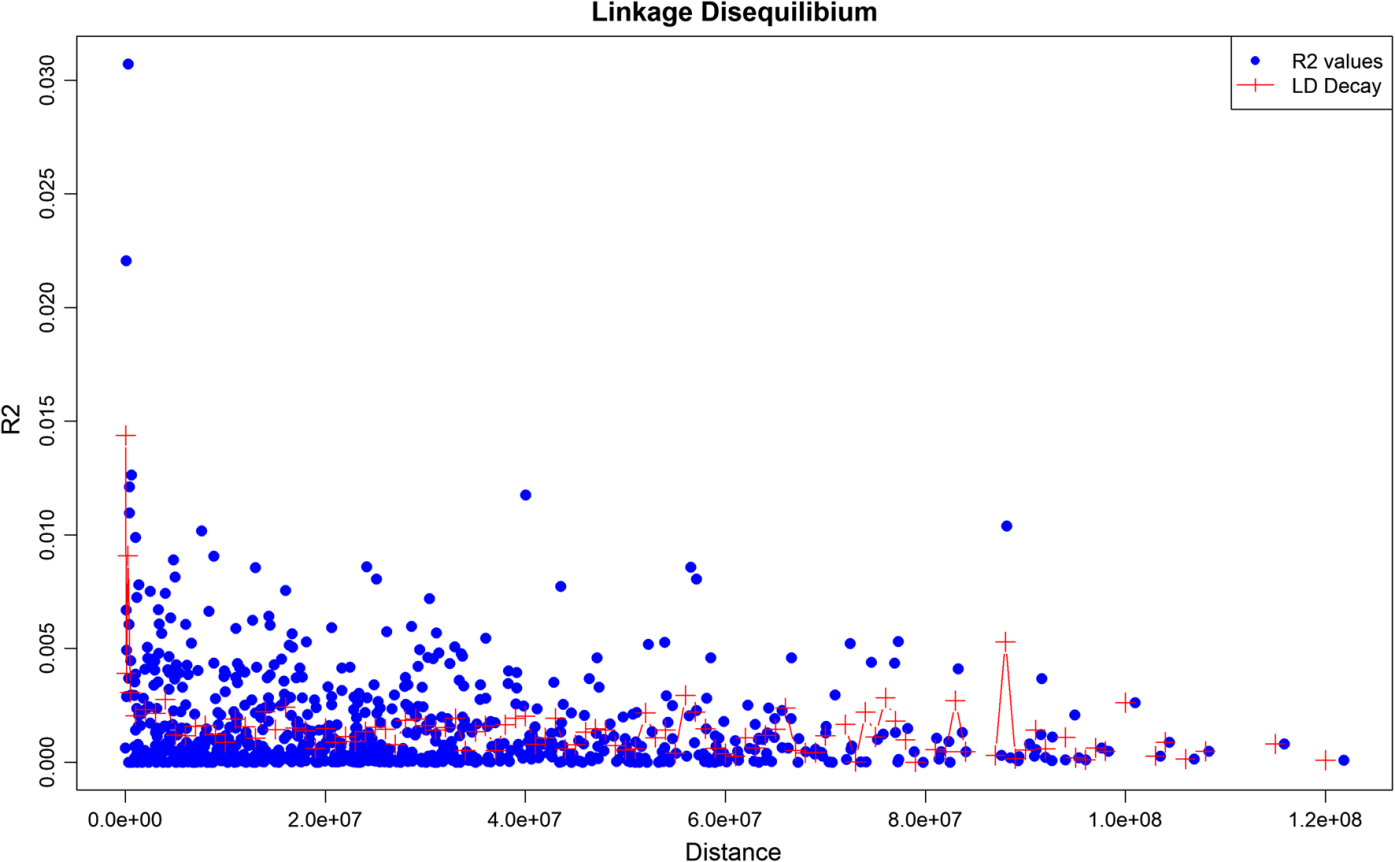
Linkage disequilibrium between the 195 SNPs of the reduced panel, calculated as *R*^2^ values for each pair of SNPs (blue dots). In red, we report the LD decay as the average in the following windows: 10–100 kb, 100–250 kb, 250 kb–1 Mb and then every 1 Mb

Pairwise LD between all SNPs included in the 195-SNP panel was estimated (Fig. 1), with values *r*^2^ ranging from 5.9 × 10^−12^ to 0.03, with an average (± SD) of 0.0016 ± 0.0024.

#### Parentage and sex assignment performances

After the estimation of LD levels in the whole dataset, an evaluation of the exclusion ability of the panel was performed based on the shared discordant homozygotes (< 1% of all SNPs). The performances were then reported as: the ability to assign true relationships correctly (sensitivity), the ability to exclude unrelated pairs correctly (specificity), the overall ability to assign a pair of individuals to its group (accuracy), the probability of being related having a positive result (positive predictive value; PPV) and of being unrelated having a negative response (negative predictive value; NPV). Performances were estimated for the cleaned dataset using the 195-SNP panel with and without the SNPs on the X chromosome (Table 1). As shown in Table 1, the panel including SNPs on the X chromosome displayed a strong ability to exclude unrelated individuals as parents, with only two false positive among the 7.5 million pairwise comparisons. However, it shows a low sensitivity, excluding a large number of pairwise comparisons among related individuals. All but one of these unassigned related pair involved at least one male. Thus, exclusion ability differed between pairs that involved males compared to female-only pairs. In fact, performing an assignment without the SNPs on the X chromosome resulted in a slightly lower specificity (four false positives instead of two) but to a much higher sensitivity (four false negatives instead of 174). This confirms the need to remove the SNPs mapped to the X chromosome in comparisons involving males, since they are uninformative and can lead to a large number of false negatives.

**Table 1:** Performance of the 195-SNP panel on the cleaned dataset using the full 195-SNP panel (left column) and using the panel without the SNPs on the sex chromosomes (right column)

	Performances including the sex chromosome markers	Performances excluding the sex chromosome markers
*Pair number*		
Related pairs	802	802
Unrelated pairs	7,551,639	7,551,639
Total number of pairs	7,552,441	7,552,441
*Assignment*		
True positive (a)	628	798
False negative (b)	174	4
False positive (c)	2	4
True negative (d)	7,551,637	7,551,635
*Diagnostic parameter*		
Sensitivity	78.30%	99.50%
Specificity	100.00%	100.00%
Positive predictive value	99.68%	99.50%
Negative predictive value	2.30408E−05	0.999999735
Accuracy	0.999976696	0.999999204

The inclusion of X-linked markers in the direct assignment led to the exclusion of a large number of related pairs, whereas several are correctly assigned using the autosomal SNPs. This is because almost all these pairs involve a male-male comparison, highlighting how these SNPs cannot be used to perform a proper hemizygous evaluation, and is consistent with the higher Pe values found for the SNPs on the sex chromosomes

The assignment efficiency was evaluated on an independent dataset from a QTL design which included the true sires, and consisting of 57,510 father-offspring combinations in the Saanen breed and 95,175 in the Alpine breed. For 752 combinations in the Saanen breed and 1143 in the Alpine breed, no Mendelian inheritance conflicts were found, which corresponds to 93 and 97% of the total number of offspring, respectively for the Saanen and Alpine breeds. The LOD score was positive for 840 combinations in the Saanen breed and 1222 in the Alpine breed, which is more than the number of single father-offspring combinations. The posterior probability reached a threshold of 99% for all the offspring in the Saanen breed and for 1173 pairs (99.8% of the total number of offspring) in the Alpine breed. Among the 1175 females of the Alpine breed and the 810 females of the Saanen breed, all were assigned to their true sires if these were included, which corresponded to a power of 100%. The sensitivity is also 100% since no females were assigned to an incorrect sire, whether the true sire was included or not.

The MAF for each SNP on the X chromosome (*N* = 19) and each population (*N* = 106) ranged from 0.10 to 0.50 (mean = 0.35). Based on these MAF, the probability of being homozygous simultaneously at the 19 SNPs was estimated. On average for the 106 populations, this probability reached 2.9 × 10^−5^, which indicates that a female can be detected with a very high reliability, using our SNP panel. This probability varied according to the population but remained very low regardless of the population: the best result was obtained for the Barki breed (probability = 3.6 × 10^−6^) and the worst for the Palmera breed (probability = 3.9 × 10^−4^).

## Conclusions

Our findings suggest that the panel of SNPs identified here is suitable and readily applicable for parentage and sex assessment and assignment for a large number of worldwide goat breeds. In addition, this panel was validated on a large, independent dataset that includes French Saanen and Alpine goats. Although this tool could be of great value for breeding and genetic variability management, the on-field application is still limited due to the high cost of the technology which, compared to the value of the individuals, is still unaffordable. However, the fast and strong improvement of genotyping technologies will probably allow cost-effective genotyping of small sets of SNPs in the near future. This would provide the opportunity to apply this technology to goats and allow a better management of the biodiversity and genetic improvement of this economically important species, especially in marginal areas of the world.

## Additional files

**Additional file 1: Table S1.** Description of the populations and performances of the final 195-SNP panel with these populations. Description: The table contain the population code (as BreedCode_CountryCode), the full breed name, the sample size for both procedures, the number of parent-offspring pairs identified from the genotypes and the performances of the final 195-SNP panel on each population as: (i) cumulative probability of exclusion (Pe, shown as -log10(Pe) to increase readability), (ii) cumulative probability of inclusion (Pi, shown as -log10(Pi)/10 to increase readability) and (iii) the minor allele frequencies.

**Additional file 2: Table S2.** Description of the 195 SNPs resulting from the selection. Description: The table includes the details for the 195 SNPs identified by both relationship- and MAF-based procedures. Column from A to D include SNP ID, in which panel they were identified (50-SNPs panel from the MAF based procedure only, the 147-SNPs panel from the relationship-based procedure only or identified by both approaches), their chromosome and their physical position. Columns from F to K include chromosome statistics of the identified SNPs, such as: chromosome ID, initial number of SNPs on the chromosome, final number of SNPs retained on the chromosome, percentage of SNPs retained after reduction, chromosome sizes as base pair count and tens of Mb. The bar plot shows the distribution of SNPs regardless of chromosome size for the different chromosomes.

**Additional file 3: Figure S1.** Number of SNPs with no exclusion probability (a) and with inclusion probability = 1 (b) by number of breeds.
