# Supplementary material for: Functional SNP panel for parentage assessment and assignment in worldwide goat breeds
Source: Genet Sel Evol. 2018 Nov 19;50:55. doi: 10.1186/s12711-018-0423-9 (PMC6240953; doi:10.1186/s12711-018-0423-9)
Supplement: Supplementary file 3 — Additional file 3: Figure S1. Number of SNPs with no exclusion probability (a) and with inclusion probability = 1 (b) by number of breeds. [file 12711_2018_423_MOESM3_ESM.docx]

**Supplementary material for**: “Functional SNP panel for parentage assessment and assignment in worldwide goat breeds”

Andrea Talenti, Isabelle Palhière, Flavie Tortereau, Giulio Pagnacco, Alessandra Stella, Ezequiel L. Nicolazzi, Paola Crepaldi, Gwenola Tosser-Klopp and ADAPTmap Consortium

**Figure S1**: Number of SNPs with no exclusion probability (A) and with inclusion probability = 1 by number of breeds.
